# Comparative genomic analysis of novel conserved peptide upstream open reading frames in *Drosophila melanogaster *and other dipteran species

**DOI:** 10.1186/1471-2164-9-61

**Published:** 2008-02-01

**Authors:** Celine A Hayden, Giovanni Bosco

**Affiliations:** 1 Department of Molecular and Cellular Biology, University of Arizona, Tucson, AZ 85721, USA

## Abstract

**Background:**

Upstream open reading frames (uORFs) are elements found in the 5'-region of an mRNA transcript, capable of regulating protein production of the largest, or major ORF (mORF), and impacting organismal development and growth in fungi, plants, and animals. In Drosophila, approximately 40% of transcripts contain upstream start codons (uAUGs) but there is little evidence that these are translated and affect their associated mORF.

**Results:**

Analyzing 19,389 *Drosophila melanogaster *transcript annotations and 666,153 dipteran EST sequences we have identified 44 putative conserved peptide uORFs (CPuORFs) in *Drosophila melanogaster *that show evidence of negative selection, and therefore are likely to be translated. Transcripts with CPuORFs constitute approximately 0.3% of the total number of transcripts, a similar frequency to the Arabidopsis genome, and have a mean length of 70 amino acids, much larger than the mean length of plant CPuORFs (40 amino acids). There is a statistically significant clustering of CPuORFs at cytological band 57 (p = 10^-5^), a phenomenon that has never been described for uORFs. Based on GO term and Interpro domain analyses, genes in the uORF dataset show a higher frequency of ORFs implicated in mitochondrial import than the genome-wide frequency (p < 0.01) as well as methyltransferases (p < 0.02).

**Conclusion:**

Based on these data, it is clear that Drosophila contain putative CPuORFs at frequencies similar to those found in plants. They are distinguished, however, by the type of mORF they tend to associate with, Drosophila CPuORFs preferentially occurring in transcripts encoding mitochondrial proteins and methyltransferases. This provides a basis for the study of CPuORFs and their putative regulatory role in mitochondrial function and disease.

## Background

It is becoming increasingly clear that controlling protein levels post-transcriptionally is an important mechanism for growth and development in eukaryotic cells. Upstream start codons (uAUGs), AUGs found 5' of the longest, or major, open reading frame (mORF), occur in 20–50% of eukaryotic mRNAs of a given genome [1-5]. When translation is initiated at a uAUG, these upstream ORFs (uORFs) can affect the protein level of the mORF with serious biological consequences. uORFs can regulate mORF protein production in response to starvation conditions [6], polyamine concentrations [7,8], and sucrose levels in the cell [9]. For example, the yeast *General Control Nondepressible 4 *(*GCN4*) transcript contains multiple uORFs that differentially regulate the protein level of a transcription factor-encoding mORF under starvation and non-starvation conditions. In turn, the protein produced from the mORF, the GCN4 protein, is essential to the transcriptional activation of some 40 genes involved in amino acid biosynthesis [6]. Because uORFs influence the levels of mORF protein, it is not surprising that disruption of the uAUG can lead to human disease such as thrombocythemia [10], a disease which is thought to arise as a result of increased mORF protein product, thrombopoietin (TPO). In addition, uAUGs occur in transcripts coding for oncogenes more frequently than other mammalian transcripts [11]. Indeed, oncogenes *Mdm2 *[12], *her-2 *[13], *MYEOV *[14], *Bcl-2 *[15], and *SCL *[16], all contain uORFs that affect the level of oncoproteins produced.

Potentially thousands of genes are regulated via uORFs, but there are no demonstrated examples of uORFs affecting mORF protein production in *Drosophila *or other insect species. Several uORF-containing genes have been well studied in fungi, plants, and mammals [17] and genome-wide searches of conserved uORFs have been conducted using fungal, mammalian and plant transcripts [4,18-21]. Given the examples found in other eukaryotic species, it is plausible that uORFs fill a regulatory role in the arthropod lineage as well.

There is some evidence that regulatory uORFs may occur in insect species. Firstly, a Drosophila gene coding for a putative mannosyl transferase contains a uORF-mORF pair that seems to be evolutionarily conserved in insects [19]. Secondly, there are several examples of Drosophila dicistronic transcripts in which the first open reading frame could be regulatory to the second [22-24]. However, polycistronic transcripts do not all code for putative uORFs; many transcripts defined as polycistronic are initially transcribed as pre-mRNA with two or more ORFs, but are subsequently processed into separate monocistronic transcripts [25]. For this reason, we prefer to use the terminology 'uORF' to refer to an ORF (a) which is upstream of a mORF on a single mature mRNA, and (b) which is itself translated as a polypeptide distinct from protein translated from a mORF. In addition, polycistronic transcripts that are not processed into separate mRNA molecules are at times part of this uORF/mORF classification. The computational identification of dicistronic transcripts by Misra et al [22] resulted in the reannotation of 31 gene models, some of which may contain conserved uORF-mORF pairs. However, their search was limited to polycistronic transcripts with ORFs greater than 50 a.a., and it is known that uORF peptides as short as 6 a.a. can regulate mORF translation in mammals [26]. Their analysis also discarded overlapping ORFs, some of which are important for the regulation of mORFs [27].

To identify transcripts with uORFs that are likely to be translated, we took a comparative genomics approach using *D. melanogaster *transcript annotations, *Anopheles gambiae *transcript annotations, and dipteran expressed sequence tags (ESTs). Using this approach, we determined the prevalence, diversity, and genomic clustering of CPuORFs under negative selection in dipteran genomes and compared these findings to those reported for the plant lineage.

## Results and Discussion

### Identification of conserved peptide uORFs in *D. melanogaster*

To determine the prevalence of uORFs most likely to be translated, *Drosophila melanogaster *release 4.3 transcript sequences (19,389) were used to identify the largest, or major, ORF (mORF). Of these, 13,746 contain unique Flybase gene numbers, 5,851 of which contain one or more AUGs upstream of the mORF. This suggests that 43% of *Drosophila *mORF proteins could be affected in their expression level by translated uORFs. Our calculated percentage is slightly lower than previously reported *Drosophila *uAUG frequencies [2], but this discrepancy can be explained by the smaller dataset used in the previous study.

Putative dipteran homologs were found by comparing *D. melanogaster *mORFs to 666,153 NCBI ESTs using tBLASTn. Many of the EST sequences contained truncated uORF and mORF sequences, therefore the search was limited to species that diverged from *D. melanogaster *more than 15 Mya (non-melanogaster group species; AAA: 12 Drosophila Genomes Website) [28,29], to increase detection of negative selection acting on short protein sequences. For each pair of homologs, global alignment of uORFs identified candidate CPuORFs and *K*_*a*_*/K*_*s *_ratios were used to further verify evolutionary conservation of the uORF amino acid sequence. In addition, Flybase transcript annotations were used to discard any genes in which the putative CPuORF was fused to the mORF in any given transcript splice variant.

*K*_*a*_*/K*_*s *_ratios < 1 indicate that a sequence is under negative selection, *K*_*a*_*/K*_*s *_ratios close to 1 imply that the sequence is undergoing drift, and *K*_*a*_*/K*_*s*_ratios > 1 suggest that the sequence is under positive selection. We found a total of 44 CPuORFs with a *K*_*a*_*/K*_*s*_ratio significantly less than one (Table 1; Additional File 1). Importantly, our *K*_*a*_*/K*_*s *_ratio analysis distinguishes between high-scoring amino acid alignments that reflect conservation of nucleotide sequences versus alignments that reflect true evolutionary conservation of the amino acid sequence, and therefore are good indicators of translation.

**Table 1:** *K*_*a*_*/K*_*s *_values of uORF and associated mORFs correlated to most distantly related organism containing uORF-mORF association in an EST

**CG identifier**	**uORF**	**mORF**	**Most distantly related organism (NCBI accession #), most closely shared taxonomic classification with *D. melanogaster*^a^**
CG18624	0.11****	0.06****	*Boomic*^b ^(CV448373), Arthropoda
CG12664	0.26**	0.15****	*Drovir *(EB568517), Drosophila
CG12788/CG17767	0.32****	0.28****	*Anogam *(CD747020), Diptera
CG33713/CG33714	0.06****	0.13***	*Carmae *(DW250045), Pancrustacea
CG3240	0.11****	0.11****	*Dromoj *(EB613491), Drosophila
CG9960/CG9958	0.02****	0.07****	*Dapmag *(DY0373460), Pancrustacea
CG31917	0.00****	0.09****	*Bommor *(DY230769), Endopterygota
CG31919/CG33995	0.10**	0.31*	*Glomor *(DV616490), Schizophora
CG18042	0.01****	0.29*	*Bommor *(AU003981), Endopterygota
CG7400	0.10*	0.06****	*Dropse *(DR124033), Sophophora
CG16974	0.00**	0.14****	*Dropse *(DR133486), Sophophora
CG4824	0.04***	0.17***	*Dropse *(DR131819), Sophophora
CG17325	0.08****	0.07****	*Drogri *(EB611588), Drosophila
CG10570	0.28**	0.19****	*Drogri *(EB601583), Drosophila
CG11508	0.13****	0.54**	*Glomor *(DV620389), Schizophora
CG8026	0.31*	0.04****	*Drogri *(EB598775), Drosophila
CG17759 (uORF2)	0.33*	0.02****	*Dromoj *(EB608824), Drosophila
CG33671/CG33672	0.07****	0.14****	*Apimel *(DB747777), Endopterygota
CG6191	0.13**	0.05***	*Drogri *(EB625487), Drosophila
CG30100	0.08****	0.09****	*Ixosca *(DN974785), Arthropoda
CG17725	0.00*	0.06****	*Drowil *(EB488086), Sophophora
CG5469	0.10****	0.07****	*Aedaeg *(EB099927), Diptera
CG33786/CG33785	0.03**	0.16****	*Bommor *(BB992822), Endopterygota
CG9865 (uORF1)	0.12****	0.30****	*Drowil *(EB454746), Sophophora
CG9865 (uORF2)	0.07****	0.30****	*Aedaeg *(DV278474), Diptera
CG9865 (uORF3)	0.04****	0.30****	*Acypis *(CV847404), Neoptera
CG9878	0.30**	0.04****	*Ixosca *(AF483733), Arthropoda
CG30290	0.00****	0.05*	*Carmae *(DY308116), Pancrustacea
CG12016	0.12****	0.12****	*Acypis *(CN762015), Neoptera
CG32573	0.42***	0.19****	*Drowil *(EB501531), Sophophora
CG11989	0.04****	0.01****	*Myzper *(EE261505), Neoptera
CG7869	0.09****	0.12****	*Dromoj *(EB608881), Drosophila
CG7628	0.10**	0.03****	*Glomor *(DV612431), Schizophora
CG9666	0.29****	0.04****	*Artfra *(BQ605225), Pancrustacea
CG2128	0.24****	0.00****	*Bommor *(BY914486), Endopterygota
CG9288	0.16****	0.17****	*Aedaeg *(DV427990), Diptera
CG9924	0.08*	0.12****	*Drovir *(EB563704), Drosophila
CG31241	0.23****	0.00*	*Dromoj *(EB603524), Drosophila
CG31178	0.31***	0.33**	*Drovir *(EB564030), Drosophila
CG7071/CG34131	0.08****	0.20****	*Lutlon *(AM099995), Diptera
CG10238	0.29****	0.12****	*Taegut *(DV959401), Coelomata
CG5116	0.15**	0.16****	*Drowil *(EB489685), Sophophora
CG14550	0.13*	0.21****	*Bommor *(CK562143), Endopterygota
CG7950	0.35****	0.04****	*Myzper *(EE263186), Neoptera

^a ^*D. melanogaster *taxonomic classification as described by NCBI

^b ^Abbreviations: Boomic, *Boophilus microplus*; *Drovir, Drosophila virilis*; *Anogam, Anopheles gambiae*; *Carmae, Carcinus maenas*; *Dromoj, Drosophila mojavensis*; *Dapmag, Daphnia magna*; *Bommor, Bombyx mori*; *Glomor, Glossina morsitans*; *Dropse, Drosophila pseudoobscura*; *Drovir, Drosophila virilis*; *Drogri, Drosophila grimshawi*; *Apimel, Apis mellifera*; *Ixosca, Ixodes scapularis*; *Drowil, Drosophila willistoni*; *Aedaeg, Aedes aegypti*; *Acypis, Acyrthosiphon pisum*; *Myzper, Myzus persicae*; *Artfra, Artemia franciscana*; *Lutlon, Lutzomyia longipalpis*; *Taepyg, Taeniopygia guttata*

* p-value < 0.05; H_0_: *K*_*a*_*/K*_*s *_= 1, H_*A*_: *K*_*a*_/*K*_*s *_< 1

** p-value < 0.01

*** p-value < 0.001

****p-value < 0.0001

Another indicator of translation is start codon context. Based on nucleotide frequencies of sequences surrounding mORFs, it is predicted that the *Drosophila *optimal consensus sequence is CAaaAUGg [2,30], but no functional experiments have been conducted in insects to validate the strength of this initation context. Therefore, although the predominant CPuORF start context (AAaaAUGa) seems to be weaker than the predominant mORF context, it remains to be determined whether ribosomes initiate efficiently at the uORF AUG. It is also quite likely that initiation of some CPuORFs is dependent upon cellular conditions, as has been shown in various genes [6,31], leading to regulation of mORF protein levels.

A number of uORF-mORF pairs were used as positive controls for the modified uORF-Finder program. In a previous study, CG9865 was shown to contain a putative uORF-mORF pair that has been conserved among distantly related insect species [19]. This gene was identified by our analysis, therefore validating our approach. *Drosophila Tat-like *(*DTL*), a gene containing a uORF with amino acid similarity in *D. melanogaster *and *D. pseudoobscura *[24] was also found by the uORF-Finder program. A third gene identified by our analysis, CG10238, is a bicistronic transcript encoding the small and large subunit of Molybdopterin synthase 2 (MOCS2) [23]. It is well conserved across distantly related eukaryotic species (see Additional File 2). In addition, 5 of the 31 dicistronic genes described by Misra et al [22] were shown to contain CPuORFs (Table 2; denoted by Misra and colleagues as CG33071ORFA-CG33071ORFB, Tim9b-CG12788, CG33009ORFA-CG33009ORFB, CG33005ORFA-CG33005ORFB, and *snapin*-CG9960, but subsequently renamed CG33713-CG33714, CG12788-CG17767, CG33671-CG33672, CG33786-CG33785, and CG9960-CG9958, respectively). Many of the dicistronic transcripts identified by Misra et al [22] are transcripts with ORF pairs that are not well conserved among the *Drosophila *species. For example, the mei217-mei218uAUG is not conserved in any of the 11 other sequenced *Drosophila *genomes (UCSC *D. melanogaster *genome browser) [32], therefore it is not surprising that a number of the dicistronic genes were not identified by the uORF-Finder program. Additionally, it is likely that neither the *D. melanogaster *annotations nor the dipteran ESTs are representative of the complete transcript population within each species due to the incomplete annotation of 5' transcription start sites [33], and incomplete coverage of the genomes by ESTs.

**Table 2:** Cytological distribution and peptide length of putative CPuORFs in *Drosophila melanogaster*

**Flybase transcript identifier and uORF number (FBtrXXXXX_#)**	**CG identifier**	**Cytological gene location**	**uORF length (a.a.)**
FBtr0071140_1	CG18624	7C2-7C2	54
FBtr0071349_3	CG12664	8C11-8C13	41
FBtr0074767_3	CG12788/CG17767^b^	18D3-18D7	117
FBtr0077227_1	CG33713/CG33714^b^	19F4-19F4	90
FBtr0077747_1	CG3240	23A1-23A1	179
FBtr0077737_2	CG9960/CG9958^b^	23A3-23A3	134
FBtr0079037_2	CG31917	25C1-25C1	73
FBtr0079006_1	CG31919/CG33995^b^	25C1-25C1	44
FBtr0079695_3	CG18042	29D4-29D5	85
FBtr0080133_1	CG7400	31F4-31F5	20
FBtr0080489_1	CG16974	34A8-34A8	21
FBtr0080803_5	CG4824	35E2-35E2	44
FBtr0081102_1	CG17325	37A4-37A5	48
FBtr0081122_2	CG10570	37A4	50
FBtr0088817_5	CG11508	44B3-44B3	150
FBtr0088610_3	CG8026	45B3-45B3	48
FBtr0087829_3	CG17759	49B8-49B9	31
FBtr0091650_2	CG33671/CG33672^b^	49B10-49B10	86
FBtr0087678_3	CG6191	50B3-50B4	21
FBtr0087140_1	CG30100	53B1-53B1	70
FBtr0086701_1	CG17725	55D3-55D3	27
FBtr0086654_7	CG5469	55E5-55E5	121
FBtr0091786_1	CG33786/CG33785^b^	57A8-57A9	108
FBtr0071680_7	CG9865^a ^(uORF1)	57F7-57F7	65
FBtr0071680_5	CG9865^a ^(uORF2)	57F7-57F7	84
FBtr0071680_4	CG9865^a ^(uORF3)	57F7-57F7	76
FBtr0071676_1	CG9878	57F8-57F8	65
FBtr0071672_1	CG30290	57F8-57F9	94
FBtr0073063_4	CG12016	63D1-63D1	81
FBtr0074315_3	CG32573	14F5-14F5	109
FBtr0076348_2	CG11989	67D2-67D2	50
FBtr0076203_3	CG7869	68A4-68A4	68
FBtr0076213_1	CG7628	68A7-68A8	18
FBtr0074991_5	CG9666	76A3-76A3	129
FBtr0078767_1	CG2128	83A4-83A4	38
FBtr0082829_3	CG9288	87F13-87F13	80
FBtr0082871_2	CG9924	88A3-88A4	25
FBtr0083570_4	CG31241	90F11-90F11	178
FBtr0084138_3	CG31178	93F14-93F14	40
FBtr0084211_1	CG7071/CG34131^b^	94A6-94A6	157
FBtr0084782_2	CG10238	96C1-96C1	90
FBtr0084877_1	CG5116	96E2-96E2	15
FBtr0084974_2	CG14550	96F10-96F10	111
FBtr0085563_1	CG7950	99D3-99D3	111

^a ^Gene with multiple CPuORFs in the same 5'UTR

^b ^Different gene identifiers annotated as producing the same transcript; the first CG identifier predicts the translation of the mORF and the second CG identifier predicts the translation of the uORF.

Initially, 41 genes and 43 uORFs showed evidence of mild to strong purifying selection (*K*_*a*_*/K*_*s *_ratio significantly < 1), and an additional gene with one uORF was detected during subsequent duplication analysis (see below). The proportion of genes in the *Drosophila *genome showing evidence of CPuORFs is approximately 0.3% (42 genes out of 14,040 genes), which is similar to the frequency predicted for the Arabidopsis genome (0.4–0.5%) [19]. The present study likely underestimates the prevalence of CPuORFs due to incomplete EST resources and potentially misannotated 5' regions in *D. melanogaster*.

Consistent with calculated *K*_*a*_*/K*_*s*_values, the majority of CPuORFs with a low *K*_*a*_*/K*_*s *_ratio are present in lineages beyond the Drosophilidae (Table 1) and therefore have been conserved more than 40 My (Assembly/Alignment/Annotation of 12 Drosophila species) [28,29]. Those uORFs that exhibit a low *K*_*a*_*/K*_*s *_ratio but are only found within Drosophila species may represent uORFs that have recently emerged within the Drosophila lineage but are nonetheless under mild to strong selection pressures.

### Insect CPuORFs are longer in average length than plant CPuORFs

**Figure 1:**
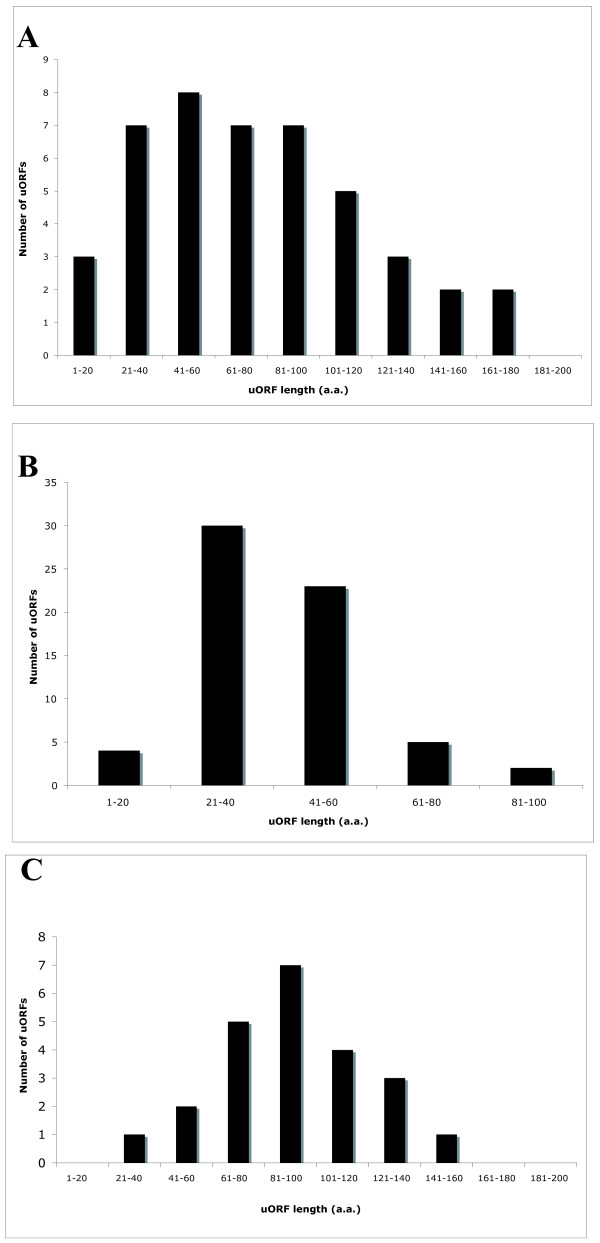
**Conserved peptide uORF length distribution**. A. A total of 44 CPuORFs identified in *Drosophila melanogaster*, B. CPuORFs in *Arabidopsis thaliana *as described by Hayden and Jorgensen [19], C. CPuORFs conserved between *D. melanogaster *and non-*Brachycera *species.

Two studies have shown that the length of a uORF can influence the ability of a ribosome to reinitiate scanning and translation initiation at a mORF [34,35]. The plant and mammalian cell systems used in these studies show that reinitiation at a downstream AUG is generally more efficient in the presence of shorter uORFs, and in plant protoplasts reinitiation drops sharply in constructs containing uORFs longer than 34 amino acids. Both studies were carried out using viral components, and as such it is not clear whether these observations extend to mRNAs in a native eukaryotic cellular environment. Nonetheless, uORF length could play an important role in the regulation of mORFs, therefore we analyzed Drosophila CPuORFs in terms of their amino acid lengths. Initial characterization of the 44 putative CPuORFs under negative selection reveals a wide distribution of lengths, ranging from 15 to 179 amino acids (Table 2, Figure 1A).

To date, most, if not all, functionally characterized uORFs are smaller than 100 amino acids, but more than one fourth (12/44) of *D. melanogaster *CPuORFs are above this size. In general, Drosophila CPuORFs seem to be larger than those found in plants. While 83% of Arabidopsis CPuORFs are between 21 and 60 amino acids in length (mean of 40 amino acids ± 16 standard deviation; Figure 1B), the Drosophila uORF length distribution peaks between 41 and 80 amino acids (mean of 76 amino acids ± 44; Figure 1A). These plant and insect datasets were not generated by comparing species with the same evolutionary distance but a more convincing comparison can be made by analyzing uORFs that have been conserved over more than 200 My: between Arabidopsis and rice, and between Drosophila and non-*Brachycera *lineages (e.g. *Anopheles*). The Arabidopsis distribution peak remains essentially unchanged under these restrictions (mean of 39 amino acids ± 13), whereas the distribution of Drosophila uORFs peaks at an even greater length, 81–100 amino acids (mean of 92 amino acids ± 29; Figure 1C). Longer uORF lengths in Drosophila may reflect a need for stronger suppression of mORF translation than in plants, consistent with the observations of the above-mentioned cell culture studies. Alternatively, insect cells may exhibit more efficient reinitiation resulting in a requirement for longer uORFs to attenuate mORF translation.

### Physical mapping reveals clustering of CPuORFs independently of gene duplication

In insect and mammalian genomes, clusters of closely related genes can sometimes occur, such as the Hox gene clusters [36]. To determine whether genes with uORFs cluster in certain parts of the genome, the 44 uORFs were placed on the *D. melanogaster *cytological map (Table 1) and compared to a random distribution (Methods). uORF frequencies were not statistically different from a randomly generated dataset except for a cluster of 6 uORFs residing on band 57 (p-value = 10^-5^), five of which fall on a much smaller segment of the chromosome, band 57F. Upon closer examination, some of these uORFs may have arisen as a result of tandem duplications; one uORF found in the CG30290 transcript as well as two uORFs found in the CG9865 transcript (uORF1 and uORF3) all contain twin CX_9_C motifs. Interestingly, the observed clustering is not dependent upon the putative duplication events of CX_9_C motif-containing uORFs. Eliminating the duplication bias by collapsing CX_9_C-containing uORFs to one representative, clustering is still statistically significant, with 4 uORFs on cytological band 57 (p-value = 0.004) and 3 uORFs on band 57F (p-value = 0.0002). Therefore, the data suggest that there is a preponderance of both clustering and duplicate retention of uORFs on band 57. Clustering at this region could be an indicator of chromatin interactions at this site that could mediate CPuORF regulation.

The twin CX_9_C motif is an integral part of coiled-coil helix, coiled-coil helix (CHCH) domains, a domain previously implicated in uORF-mORF associations in group 8 plant uORFs [19]. In fact, the group 8-like *Drosophila *uORF member described in the plant study is uORF3 of CG9865. It is interesting to note that the plant group 8 uORF has consistently lost its duplicate copy during both recent and ancient polyploidy events whereas the Drosophila group 8 putative homologue may be retaining its duplicates. Different duplication retention histories could indicate that twin CX_9_C motif-containing ORFs play different roles in plants and animals.

### CPuORF-mORF pair duplicate retention is low within *Drosophila melanogaster*

To determine whether there has been retention of uORF-mORF pair duplicates within the *Drosophila *genome itself, the 41 mORFs with strongly conserved uORFs were compared to the *D. melanogaster *transcriptome. A single gene, CG17325 showed evidence of a duplicate copy, CG10570, in which the uORF-mORF pair is conserved (See Additional File 3). CG10570 was not detected by our program due to the short length of its mORF (< 100 amino acids), therefore this gene was added to our list of CPuORFs following our duplication analysis (Tables 1 and 2). CG17325 and CG10570 reside adjacent to one another on chromosome 2, band 37A4-A5, and are transcribed on opposite strands away from one another. The close proximity of the genes suggests a segmental duplication gave rise to the two genes, both of which are conserved throughout the *Drosophila *lineage and exhibit a *K*_*a*_*/K*_*s *_ratio < 0.28 (Table 1). This duplication presumably occurred more than 40 Mya since both loci are present in *D. melanogaster*, *D. grimshawi*, and *D. virilis*. Unlike the extensive uORF-mORF duplication retention history of the Arabidopsis genome, CG17325 and CG10570 were the only example of gene duplicate retention in *Drosophila*.

### GO term and protein domain analysis suggest a link between CPuORFs and both mitochondrial proteins and methyltransferases

Further differences between plant and insect CPuORFs were observed following gene ontology (GO) term analysis. GO term frequencies in the *D. melanogaster *genome were compared to frequencies in the insect uORF dataset to look for overrepresentation of terms. P-values were determined using the Bonferroni correction method, a method that accounts for multiple comparisons and calculates a conservative p-value. Also, the recent tandem duplicate (see above) was not included in the analysis to eliminate bias from recent duplication events. Because GO terms have been assigned to all ORFs found in bicistronic transcripts, GO terms were extracted for both uORF and mORF gene identifiers, designated hereafter as the uORF dataset (41 mORFs and 7 uORFs). This analysis differs from previous analyses in plants; it not only identifies 1) classes of mORF proteins that tend to associate with CPuORFs, but it also identifies 2) ORFs that preferentially associate with other ORFs on a single transcript. In plants, a large proportion of CPuORFs associate with mORFs encoding transcription factors, however this trend was not observed in insects. Instead, mORF proteins showing evidence of N-methyltransferase activity (GO term for CG9666 and CG9960 mORFs; Table 3) tend to associate with CPuORFs (p = 0.02). This methyltransferase activity may act on DNA or RNA, since both types of Interpro domains are overrepresented in these two genes.

**Table 3:** Gene Ontology term and InterPro domain overrepresentation in uORF dataset as determined by Genemerge

**GO term or Interpro reference number**	**GO term or Interpro domain**	**Genome frequency**	**Frequency in uORF dataset**	**Bonferroni corrected P-value**
GO:0008170 (MF)	N-methyltransferase activity	10/14601	2/48^1^	0.015
GO:0045039 (BP)	protein import into mitochondrial inner membrane	6/14601	2/48^2^	0.008
IPR002296	N6 adenine-specific DNA methyltransferase, N12 class	4/14040	2/48^1^	0.004
IPR000241	Putative RNA methylase	3/14040	2/48^1^	0.002
IPR004217	Zinc finger, Tim10/DDP-type	5/14040	2/48^2^	0.006

MF, molecular function; BP, biological process

^1 ^GO term or Interpro domain observed in CG9666 and CG9960

^2 ^GO term or Interpro domain observed in CG9878 (Tim10) and CG17767 (Tim9b)

Additionally, overrepresentation of GO term 'protein import into the mitochondrial inner membrane' is driven by two proteins in the Drosophila uORF dataset, CG9878 (*Translocase of inner membrane 10*, *Tim10*) and CG17767 (*Tim9b*), which contain the Interpro Zn-finger Tim10/DDP-type domain (p = 0.01). Unlike the overrepresented methyltransferase domain, the Tim10/DDP-type domain is not limited to the mORFs, but appears in either the uORF or mORF, demonstrating that these ORFs show a preference for associating with other ORFs in a transcript. Specifically, Tim10 is encoded by the mORF of its transcript while Tim9b is encoded by the uORF. This does not imply that Tim9b does not act as a regulatory uORF, however. Tim9b may act both as a chaperone in the intermembrane space, as well as a regulatory element controlling the translation of its associated mORF.

In support of a model in which mitochondrial proteins preferentially associate with other ORFs on a single transcript, a further connection to the mitochondrial inner membrane is found when examining other genes in the uORF dataset. The CG8026 mORF encodes a putative mitochondrial folate transport protein [37,38] (Table 4). Interestingly, this trend may extend to the mammalian lineage, exemplified by the human *Uncoupling protein 2 *(*UCP2*) mORF, a putative inner mitochondrial membrane transporter. The *UCP2 *mORF is not only associated with what appears to be a CPuORF, but it is regulated by its uORF in a glutamine-dependent manner [39]. *B-cell lymphoma 2 *(*BCL-2*) is another mammalian oncogene that produces a protein from its mORF, BCL-2, which is localized to mitochondria [40] and is associated with a functional uORF [15].

**Table 4:** Predicted function and biological processes of uORF-mORF pairs in *Drosophila*

**CG identifier**	**Gene name synonyms^a^**	**Inferred function^a^**	**Inferred biological process^a^**	**Supporting evidence**
CG18624		Putative NADH dehydrogenase	Mitochondrial electron transport	Pfam domain; GO term designation
CG12664	*ld14, fend*^b^	Unknown	Neuromuscular development	[61, 62]
CG12788/CG17767^c^	*Tim9b*^b ^(uORF)	Mitochondrial inner membrane translocase subunit (uORF)	Transport across mitochondrial inner membrane (uORF)	Interpro domain
CG33713/CG33714^c^		Acyl-CoA binding (mORF) RNA binding (uORF)	Unknown	Interpro domain
CG3240	*Rad1*^b^	Putative 3'->5' exonuclease activity	DNA repair	[63, 64]
CG9960/CG9958^c^	*snapin *(uORF)	Putative methyltransferase (mORF) Putative Biogenesis of Lysosome-related Organelles Complex-1-like (BLOC-1-like) subunit (uORF)	Biogenesis of lysosome-related organelles (eg. melanosomes and platelet dense granules; uORF)	[65] (uORF) Interpro domain (mORF)
CG31917	*TFB5 *(uORF)	Putative TFIIH subunit (uORF)	Transcription and DNA repair (uORF)	[66, 67]; Interpro domain
CG31919/CG33995^c^		Ankyrin repeat, protein-protein interactions	Target of transcription factor Glial cells missing (Gcm), involved in neuronal development and function	Interpro domain; [68]
CG18042	*lmg*^b^	Putative component of Anaphase Promoting Complex (uORF)	Mitosis; Neural development (unclear whether it is the uORF, mORF or both)	[69, 70]; Flybase personal communication FBrf0125046; [71]; NCBI Conserved Domain Search
CG7400	*Fatp*^b^	Putative very-long-chain fatty acyl-CoA synthetase	Fatty acid metabolism	[72]
CG16974	Member of *LIG superfamily*^b^	Leucine-rich repeat and Immunoglobulin domain-containing protein	Unknown	[73, 74]
CG4824	*BicC*^b^	RNA binding protein	Anterior-Posterior patterning	[75–77]
CG17325		Unknown	Unknown	
CG10570		Unknown	Unknown	
CG11508	*DmSNAP50, DmPBP49*^b^	Subunit of an snRNA transcriptional activator protein	Transcription of splicing factors	[78]
CG8026		Mitochondrial carrier protein	Mitochondrial folate transport	[37, 38]
CG17759^b ^(uORF2)	*Galpha49B, Gqα *^b^	G-protein subunit	Photoreceptor signal transduction; Axonal guidance	[79–81]
CG33671/CG33672^c^		Mevalonate kinase (mORF); BolA-like protein, putative nucleic acid binding protein (uORF)	Isoprenoid production (mORF)	[82, 83]
CG6191		Unknown	Unknown	
CG30100		Translation release factor	Translation termination	GO term designation
CG17725	*Pepck*^b^	Putative phosphoenolpyruvate carboxykinase	Gluconeogenesis; Starvation; Glyceroneogenesis	[84–86]
CG5469	*Gint3*^b^	Ubiquitin regulatory X domain (UBX), putative RNA binding	Unknown	[87]; FBrf0189302
CG33786/CG33785^c^		Unknown	Translation (mORF) Transcription (uORF)	Interpro domain
CG9865^b ^(uORF1)		Putative mannosyl transferase	Unknown	Interpro domain
CG9865^b ^(uORF2)		Putative mannosyl transferase	Unknown	Interpro domain
CG9865^b ^(uORF3)		Putative mannosyl transferase	Unknown	Interpro domain
CG9878	*Tim10*^b^	Putative inner mitochondrial membrane translocase	Protein transport across mitochondrial membrane	[88]
CG30290		Putative flavoprotein enzyme	Unknown	Interpro domain
CG12016		Unknown	Unknown	
CG32573		Unknown	Unkown	
CG11989	*Ard1*^b^	Putative N-Acetyltransferase catalytic subunit	Unknown	[89]; Interpro domain
CG7869	*SuUR*^b^	DNA binding	Endoreplication	[90, 91]
CG7628		Phosphate transporter	Phosphate transport	Interpro domain
CG9666		Putative methyltransferase	Unknown	Interpro domain
CG2128	*Hdac3*^b^	Histone deacetylase	Wing development; Chromatin remodeling	[92, 93]
CG9288		Pyruvate kinase	Unknown	Interpro domain
CG9924	*Rdx*^b^	Unknown	Regulator of Hedgehog response (growth and development)	[94, 95]
CG31241	*DTL*^b^	Putative RNA methylase	Late larval development	[24]; Interpro domain
CG31178		Unknown	Unknown	
CG7071/CG34131^c^		Unknown	Unknown	
CG10238	*MOCS2*^b^	Molybdopterin synthase large subunit (mORF) and small subunit (uORF)	Production of molybdopterin; Implicated in mammalian neurological damage	[23, 96]
CG5116		Putative GTP-binding protein	Unknown	Interpro domain
CG14550		Putative phosphatidylinositol N-acetylglucosaminyltransferase subunit P (mORF); Pcc1-like transcription factor (uORF)	Unknown	Interpro domains
CG7950		Putative tRNA processing enzyme subunit (uORF)	tRNA processing (uORF)	Interpro domain

^a ^refers to mORF unless otherwise noted

^b^*fend*, Forked end; *Tim9b*, Translocase of inner membrane 9b; *Rad1*, Radiation insensitive 1; *lmg*, Lemming;*Fatp*, Fatty acid transport protein; *LIG*, Leucine-Rich Repeat and Immunoglobulin-containing protein (MacLaren et al, 2004); *BicC*, Bicaudal C; *DmSNAP50/DmPBP49*, snRNA activator protein 50/Proximal Sequence Element-Binding Protein 49;*Galpha49B*, G-protein alpha49B; *Pepck*, Phosphoenolpyruvate carboxykinase; *Gint3*, GDI interacting protein 3; *Tim10*, Translocase of inner membrane; *SuUR*, Suppressor of underreplication; *Ard1*, Arrest defective 1; *Hdac3*, Histone deacetylase 3; *Rdx*, Roadkill (Kent et al, 2006); DTL, Drosophila Tat-like; *MOCS2*, molybdopterin synthase 2

Other Drosophila genes also have potential links to the mitochondrion, such as CG18624, a putative NADH dehydrogenase that is predicted to act in mitochondrial electron transport (Table 4). Also, uORF1 of CG9865 is a putative homolog of p8Mature T-Cell Proliferation 1 (p8MTCP1), an ORF that is transcribed on the same mRNA as p13MTCP1, is targeted to mitochondria [41], and may play a role in oncogenesis [42,43]. CG9865 uORF1 has a twin CX_9_C motif, as do p8MTCP1 and other proteins targeted to mitochondria, namely yeast proteins Mitochondrial Ribosomal Protein 10 (Mrp10p) [44], Cytochrome Oxidase 19 (Cox19p) [45], Cytochrome Oxidase 17 (Cox17p) [46], and Mitochondrial intermembrane space Import and Assembly 40 (Mia40p) [47]. In humans, the twin CX_9_C motif found in Mia40p is required for import and stable accumulation of Mia40 in the intermembrane space [48]. Several genes in the uORF dataset contain ORFs with CX_9_C motifs, such as uORFs 1 and 3 of CG9865, the uORFs of CG30290 and CG9288, and the mORF of CG7950 (See Additional File 2). These open reading frames could be interacting with other ORFs on the same transcript to target them to the mitochondria or to form a stabilizing protein complex.

It is possible that these ORF associations are vestiges of ancient prokaryotic operons that originated in the mitochondrion and were transferred to the nuclear genome over time. This hypothesis runs counter to the prevailing thought that mitochondrial proteins involved in transport are generally of eukayotic origin [49]. Regardless of their origin, nuclear ORFs coding for mitochondrial proteins may maintain an association with other ORFs on a single transcript over long periods of evolutionary time for several reasons. Both ORFs may be co-regulated at the transcriptional level and be required at similar times in development, thus providing more efficient transcription of DNA. Alternatively, the uORF may be regulating expression of the mORF with important biological consequences. These possibilities are not mutually exclusive and further experimentation will be required to determine whether this energy-producing organelle is influenced by the translational regulation of uORF-mORF pairs on single transcripts.

Interestingly, the trend in animal mitochondrial ORFs was not observed in plants. Instead, plant uORFs tend to associate with mORFs encoding transcription factors [19]. Perhaps these unique characteristics reflect fundamental differences in the two eukaryotic lineages. Despite their differences, plants and animals both seem to contain uORF-mORF pairs involved in a wide range of biochemical and regulatory pathways (Table 4). There is some evidence in the literature that transcripts with uORFs can occur in similar biochemical pathways, such as genes affecting the polyamine biochemical pathway [50], but this is the exception rather than the rule and no additional examples have been born out by our analyses. To facilitate future studies of these elements, all CPuORF annotations will be submitted to Flybase.

## Conclusion

The identification and characterization of putative CPuORFs has established a knowledge base from which many hypotheses have been generated and can now be tested. CPuORFs in dipterans show similarities to their plant counterparts in terms of their prevalence within the genome and diversity of sequence, but differ in their greater average length, their genome clustering, and their preferential association with methyltransferases. In addition, the present analysis has shown a significant correlation between mitochondrially-targeted proteins and transcripts containing uORFs, an observation that could lead to important discoveries impacting our understanding of human disease. Given the wealth of genetic tools available in Drosophila, this model system is ideally suited to the basic understanding of uORF-containing transcripts and post-transcriptional regulation.

## Methods

### Identification of conserved peptide uORFs

*Drosophila melanogaster *transcript sequences, release 4.3 (19,389 sequences) were downloaded from Flybase [51], *Anopheles gambiae *transcript sequences, build 3.4 (14,127 sequences) were downloaded from Ensembl [52], and dipteran expressed sequence tags (ESTs) (666,153) were downloaded from NCBI [53] December 15, 2006. Because the melanogaster group members (includes *D. simulans*, *D. yakuba*, *D. erecta*, and *D. ananassae*) diverged from *D. melanogaster *relatively recently [28,29], their transcript sequences are of limited use in detecting strong negative selection over short sequence lengths due to the accumulation of few synonymous and non-synonymous substitutions. Therefore these species were excluded from this first comparison, as were *D. melanogaster *ESTs.

Comparative analysis of *D. melanogaster *and *A. gambiae *sequences was performed using uORF-Finder [19], a program that identifies the longest open reading frame of a transcript in the first species (defined as the mORF), finds the putative homolog in the second species, and aligns all open reading frames upstream of these homologs to identify putatively conserved uORFs. uORF-Finder was designed to compare full-length cDNA sequences from two species, therefore to accommodate a *D. melanogaster *full-length transcript-to-dipteran EST comparison, the program was modified and putative homologs in the ESTs were identified using the first 100 amino acids of the *D. melanogaster *mORFs. uORF size was also limited to 200 amino acids (no additional uORFs were found when uORF size was limited to 300 a.a.).

The presence of putative CPuORFs was established in at least three different species by either extracting the first 100 amino acids of the *D. melanogaster *mORF sequence and searching the NCBI EST database using tBLASTn for putative homologs with conserved uORF sequences, or by scanning the UCSC *D. melanogaster *genome browser and inspecting other *Drosophila *genomes for conservation of uORF start and stop codons [32]. Any putative uORF sequences that showed evidence of in-frame fusion with the mORF on the UCSC browser (in an alternative splice form, for example) were not included in the final list of CPuORF-containing transcripts.

### Calculation of K_*a*_/K_*s*_

The *K*_*a*_*/K*_*s *_ratio was determined using pairwise_kaks.PLS (version 1.7) [54] and is derived from the highest scoring BLAST homolog in the *D. melanogaster*-dipteran high scoring pairs. Both the approximate method (option -kaks yn00) and the maximum likelihood method (-kaks codeml) were used. Only the approximate method calculation is reported in Table 1 due to the typically short evolutionary distance between the organisms found in the highest scoring BLAST pairs. The Nei-Gojobori p-distance model was used to test for purifying selection (Null hypothesis *K*_*a *_= *K*_*s*_; alternate hypothesis *K*_*a *_<*K*_*s*_). MEGA4 default settings were used to run codon-based Z-test analyses [55] on highest scoring BLAST homologs.

### Cytological distribution of uORFs

To determine whether the 44 uORFs were randomly distributed along the Drosophila chromosomes relative to annotated transcript positions, a perl script was written to generate a random distribution of 44 positions along the chromosomes. Cytological positions for each CG gene identifier were extracted from *D. melanogaster *release 4.3 gene annotations [51], from which 44 positions were randomly chosen. This ensured that clustering would not simply reflect gene rich regions. The number of 'hits' within a given cytological band were tallied, and the entire process was iterated 30,000 times, providing a random distribution of 'hits' at any given band when 44 positions were picked across the entire genome. The random distributions were then used to provide a p-value for the observed number of uORFs within a given cytological band.

### Gene Ontology, Pfam domain, and Interpro domain retrieval and analysis

Over- and under-representation of Gene Ontology (GO) terms in the uORF dataset (41 mORFs and 7 uORFs with associated GO terms) versus the *D. melanogaster *genome was determined using Genemerge v.1.2 [56], a program which provides a Bonferroni-corrected p-value. Association files were derived from Gene Ontology website files (*D. melanogaster *annotation received from Flybase March 13, 2007) [57], and from the BioMart website [58] (Ensembl Gene ID, Pfam ID, and Interpro ID numbers obtained; downloaded files are based on *D. melanogaster *genome release 4.3). Description files were derived from GO term files [59] (gene_ontology.obo.zip), and from Interpro files [60].

## Abbreviations

Upstream open reading frame (uORF), Major open reading frame (mORF), Upstream start codon (uAUG), Conserved peptide upstream open reading frame (CPuORF), General Control Nondepressible 4 (GCN4), Thrombopoietin (TPO), Expressed sequence tag (EST), Drosophila Tat-like (DTL),Molybdopterin synthase 2 (MOCS2), Gene ontology (GO), Translocase of inner membrane 10 (Tim10), Translocase of inner membrane  9b (Tim9b), Uncoupling protein 2 (UCP2), B-cell lymphoma 2 (BCL-2), p8 mature T-cell proliferation(p8MTCP1), Mitochondrial Ribosomal Protein 10 (Mrp10p), Cytochrome Oxidase 19 (Cox19p), Cytochrome Oxidase 17 (Cox17p), Mitochondrial intermembrane space import and assembly 40 (Mia40p)

## Authors' contributions

CAH and GB conceived and designed the experiments. CAH carried out the analysis and drafted the manuscript. GB provided critical feedback for the final version. Both authors have read and approved the final manuscript version.

## Supplementary Material

Additional file 1 Conserved peptide uORF sequences. Conserved peptide uORF and associated mORF amino acid sequences in *Drosophila melanogaster*. Click here for file

Additional file 2 uORF and mORF sequences and alignments. Amino acid sequences and alignment of insect conserved peptide uORFs and associated mORFs. Click here for file

Additional file 3 CG17325/CG10570 sequences and alignment. Amino acid sequences and alignment of putatively duplicated *D. melanogaster *genes, CG17325 and CG10570. Click here for file
